# Preferred Spatial Frequencies for Human Face Processing Are Associated with Optimal Class Discrimination in the Machine

**DOI:** 10.1371/journal.pone.0002590

**Published:** 2008-07-02

**Authors:** Matthias S. Keil, Agata Lapedriza, David Masip, Jordi Vitria

**Affiliations:** 1 Basic Psychology Department, Faculty for Psychology, University of Barcelona (UB), Barcelona, Spain; 2 Computer Vision Center (CVC), Computer Science Department, Autonomous University of Barcelona, Bellaterra, Spain; 3 Department of Computer Science, Universitat Oberta de Catalunya, Barcelona, Spain; 4 Department of Applied Mathematics and Analysis (MAiA), University of Barcelona (UB), Barcelona, Spain; Northeastern University, United States of America

## Abstract

Psychophysical studies suggest that humans preferentially use a narrow band of low spatial frequencies for face recognition. Here we asked whether artificial face recognition systems have an improved recognition performance at the same spatial frequencies as humans. To this end, we estimated recognition performance over a large database of face images by computing three discriminability measures: Fisher Linear Discriminant Analysis, Non-Parametric Discriminant Analysis, and Mutual Information. In order to address frequency dependence, discriminabilities were measured as a function of (filtered) image size. All three measures revealed a maximum at the same image sizes, where the spatial frequency content corresponds to the psychophysical found frequencies. Our results therefore support the notion that the critical band of spatial frequencies for face recognition in humans and machines follows from inherent properties of face images, and that the use of these frequencies is associated with optimal face recognition performance.

## Introduction

Accumulating evidence supports the view that the processing of sensory information in the brain has adapted to statistical properties of sensory stimuli e.g., [15], [22]–[24], [26]. In this way, in principle the highest possible amount of information about the signal is encoded in the neuronal response [2], [21]. In reality, however, signal coding is subject to constraints, that include, for example, minimizing energy expenditure [3], [17], [19], [20], minimizing wiring costs between processing units [18], or reducing spatial and temporal redundancies in the input signal [1], [2], [4], [14], [29].

In a recent study, Keil [16] examined the statistical properties of a large number of face images by analyzing their amplitude spectra. The spectra were transformed such that the distribution of amplitudes versus spatial frequencies had maximum entropy (“whitening”). Whitened spectra revealed amplitude maxima at around 10 cycles per face, but only for the spectra of face images without external face features (i.e., hair, shoulder). This result compares well with corresponding psychophysical data, which suggest that humans process face identity preferentially in a narrow band of spatial frequency band (about 2 octaves) from 8 to 16 cycles per face [5]–[7], [12], [25], [27], [28], [30]. The study of Keil [16] thus suggests that the processing of face identity in humans adapted to the statistical properties of face stimuli. The psychophysical results, on the other hand, suggest that face identification is best at spatial frequencies around 10 cycles per face. Given this link between stimulus statistics and psychophysics, we reasoned that also artificial face recognition systems should show an optimal recognition performance at spatial frequencies situated around 8 to 16 cycles per face.

In this work we compare the quality of the different spatial frequencies to perform subject recognition task in the machine. The problem of subject recognition in computer vision consists on automatically assigning to a face image a label corresponding to the identity of the person that appears in the image. For this aim we usually have a set of training data from where we learn this task. Thus, the training face images are labelled according to the subject, belonging to the same class all the images obtained from the same person. This study aims to satisfy three goals: (i) To analyze the data distribution of the different spatial frequencies representations and find out if there exists a relationship between the most suitable representation in the machine and the results obtained by the psychophysical studies; (ii) to give a statistical interpretation of the human visual system procedure for recognizing faces (iii) to study which is the minimal resolution that preserves the relevant information of a face to perform computational subject recognition.

In section “Materials and Methods” we justify that the best option to evaluate features quality is using discriminability measures, which will return large values when the data is appropriately distributed to perform subject recognition and low values otherwise. Thus, to perform this study we evaluated three class discriminability measures as a function of the spatial frequency content of face images to find out if there is a maximum in the same representation found with the psychophysical studies. The obtained results suggest that artificial face recognition systems should have an optimal performance when the original face images contain spatial frequencies at around 16 cycles per degree, coinciding with the stimulus statistics and psychophysics.

## Results

**Figure 1:**
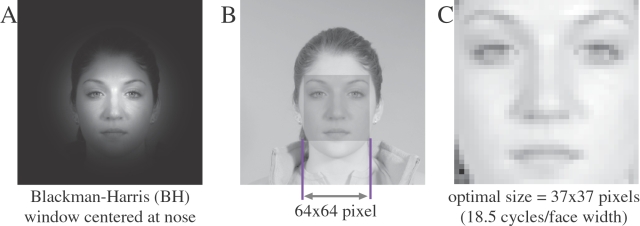
Illustration of processing steps. *(A)* External features are suppressed by centering a Blackman-Harris window at the face center (*x_no_*, *y_no_*) (indicated by a cross-hair; see methods). In this way the windowed image is obtained as shown. *(B)* The central region of each windowed face image (dark-shaded) is maintained for further processing (note that the original face image is shown here only for illustration). In this way an image with an initial size (or equivalently dimensionality) of 64×64 pixels is obtained. *(C)* Class discriminability measures are evaluated at each image size from the initial size down to 10×10 pixels. Optimal recognition performance (i.e., highest class discriminability, see Fig. 3) is obtained for images of about 37×37 pixels (here shown magnified), what corresponds to ca. 16 cycles per face width.

In the experiments, extrinsic face features (e.g., hair) were suppressed by centering a Blackman-Harris (B.H.) window at the nose (Fig. 1A and methods). To make computations feasible, spatial frequency content of face images was selected by decreasing the size of face images and applying high-pass filtering, respectively, rather than performing naive low-pass and band-pass filtering, respectively (see methods). The mentioned class discriminability measures were then computed for the down-sized images (corresponding to low-pass filtered original images), and their high-pass filtered versions (corresponding to band-pass filtered original images).

**Figure 2:**
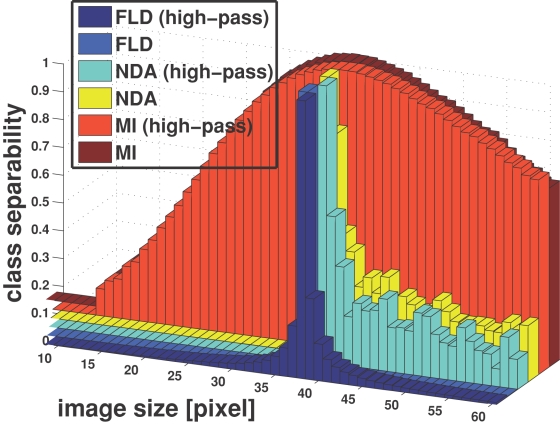
Class discriminability measures. The graphics shows normalized class discriminability measures as a function of image size (or spatial frequency). Different measures are distinguished by their color, as indicated in the figure legend: *FLD* = Fisher Linear Discriminant Analysis, *NDA* = Non-Parametric-Discriminant-Analysis,and *MI* = Mutual Information. All three measures consistently peak at around the same image size of about 37×37 pixels, corresponding to ca. 16 cycles per face width (see Fig. 2C). See text for further details.

The dependency of FLD, NDA, and MI, respectively (see Methods), on spatial frequencies (or image size) is shown in Fig. 2. Each of the three measures reveals a distinct maximum at approximately the same image size (around 37×37 pixels), what corresponds to approximately 16 cycles per face width, as illustrated by Fig. 1C. The discriminability measures have very similar dependencies on image size irrespective of applying high-pass filtering. Thus, our results suggest that class discriminability is band-pass, meaning that the lower spatial frequencies do not contribute to a good separation of classes (which can be conceived as clouds of points produced from one individual). Adopting a different viewpoint, one can also argue that decreasing image size is equivalent to reducing dimensionality, and class separation collapses beyond a certain dimension.

## Discussion

Psychophysical studies suggest that for face recognition, human observers make use of a narrow band at low spatial frequencies (8 to 16 cycles per face, bandwidth two octaves). Here we measured class discriminability, using Fisher Linear Discriminant Analysis and Non-Parametric-Discriminant-Analysis, and computing Mutual Information as a function of image size (and thus spatial frequency). These measures are used to quantify the efficiency of the different face representations to perform subject recognition in general, without depending on a specific implementation of a classifier. All three measures gave similar results for the high-pass filtered and the unfiltered face images, and revealed an unimodal distribution with a maximum at about 16 cycles per face width, which is close to the psychophysically found frequency optimum. Our results therefore support the conclusion that face representation to perform subject recognition task is optimal within a narrow band of spatial frequencies. Moreover, the presence of low spatial frequencies does not seem to compromise recognition performance.

Specifically, FLD and NDA reveal narrow peaks, which is compatible with the fact that human face discriminability of different subjects performance is best within a small band of spatial frequencies (bandwidth around two octaves, e.g., [25]). Nevertheless, MI shows a broad maximum, what may be interpreted as that recognition would still work if critical frequencies were not available. Similar observations were made in psychophysical studies [27], where it has been reported that face recognition is suboptimal in the absence of the critical frequencies. In this context, “suboptimal” means that it takes more time for subjects to recognize face identity, presumably due to a decreased signal-to-noise ratio [27].

The present study lends further support to the findings of Keil [16] in that the stimuli (i.e., face images) provide the explanation of the preference of a narrow spatial frequency band for both human and artificial face recognition. As a consequence, it is reasonable that artificial face recognition systems focus on these frequencies to achieve an optimal recognition performance, given that they are the most effective in terms of class discriminability. Because these critical spatial frequencies correspond to small image patches, a further advantage emerges through an economic use of resources for both processing and storing faces.

## Materials and Methods

### Face Images

**Figure 3:**
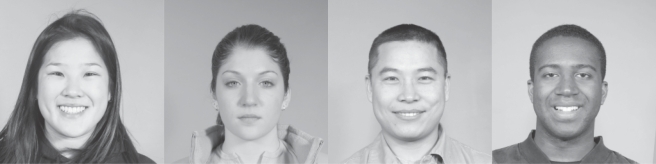
Samples from the FRGC database. The FRGC database contains male and female face images of adults from different races, with multiple photographs for each subject, different facial expressions, and different hairstyles. The faces are displayed in a fronto-parallel fashion, although some did moderately vary in posture. All faces were displayed against a uniform grey background, and illumination conditions were homogeneous and without cast shadows.

We used 868 female face images, and 868 male face images from the *Face Recognition Grand Challenge* database (FRGC, www.frvt.org/FRGC or www.bee-biometrics.org, Fig. 3) belonging to 55 different persons. We have selected all the subjects that have more than 20 images to obtain more accurate estimators of the discriminability measures. Original images (1704×2272 pixels, 24-bit true color) were adjusted for horizontal alignment of eyes, before they were down-sampled to 256×256 pixels and converted into 8-bit gray-scale. The positions of left eye (*x_le_*, *y_le_*), right eye (*x_re_*, *y_re_*), and mouth (*x_mo_*, *y_mo_*), respectively, were used to approximate the position of each face center (≈nose) as
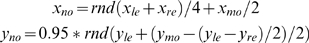
where *rnd*(*x*) denotes rounding to the nearest integer value.

### Windowing of face images

Let the features which are not part of the actual face be denoted by *external features* (e.g.,shoulder region or hair). On the other hand, *internal features* refer to the eyes, the mouth, and the nose. The presence of external features in our face images may distort recognition performance. It is thus desirable to compare results without the presence of external features. We found that a good suppression of external features could be achieved by centering a minimum 4-term Blackman-Harris window [11] at (*x_no_*, *y_no_*). The procedure is illustrated with Fig. 1A.

### Varying spatial frequency content

We adopted the following procedure to assess the frequency-dependence of face recognition. Each image was resized to continuously smaller sizes, starting with an initial size of 64×64 pixels (see Fig. 1B). We used a bilinear interpolation scheme with the Matlab function “resize” to this end (Matlab version 7.1.0.183 R14 SP3 Image Processing Toolbox, see www.mathworks.com). A down-sized image is equivalent to its low- pass filtered original image, with a cut-off frequency equivalently to the Nyquist frequency (half of pixel width or height in cycles per image). This means that the smaller image contains all spatial frequencies of the original image which are smaller or equal than the Nyquist frequency. We subsequently performed high-pass filtering of the smaller images. The latter procedure is equivalent to band-pass filtering or the original image with a narrow filter bandwidth. Notice that down-sizing reduces the dimensionality of the feature space, and saves computational time when compared to naive low-pass and high-pass filtering, respectively.

### Evaluation of Recognition Performance

The best criterion to evaluate the effectiveness of a features set to perform a concrete classification task is the Bayes error [38]. The Bayes error corresponds to the minimal probability of classification error for any given distributions [33], [34], that is, the probability that a sample is assigned to a wrong class [10]. This is the best option to evaluate features quality given that it does not depend on any specific classifier. In fact, the estimation of the Bayes error is used pattern recognition as a reference to evaluate the performance of a classification method [35].

Unfortunately, Bayes error is a theoretical definition that can not be computed if the probability densities of the data are unknown. However, upper bounds of this value can be estimated from a set of samples and these measures can be used to compare different feature sets in order to determine which is the most competitive to perform a concrete classification task. In concrete, the more effective feature set will be the one that gives a lower upper bound of the Bayes error, interpreting this value as a measure of class separability.

Different upper bounds expressions of the Bayes error can be found in the literature [13], [35], [36]. In some cases, these expressions have been used to construct discriminability measures, that is, measures that are inversely proportional to the upper bound of the error [13], [31], [35]. In this context, to find the most effective feature set among different proposals we can estimate these discriminability measures from the data and select the features with highest score.

In this work we evaluate three of the discriminablitiy measures obtained from two different upper bounds of the Bayes error. The first is the Battacharyya bound [35], which is based on scatter matrices. This upper bound yields to a class separability criteria that depends on (i) the within-class-scatter-matrix that shows the scatter of samples around the same class, and (ii) the between class scatter matrix. These measures belong to Discriminant Analysis field and depending on the computation of these scatter matrices we get a discriminability measure that assumes each class to be Gaussian distributed, or a non-parametric approach. Both computations are considered in this work and described in section “**Discriminant Analysis**”. On the other hand, we consider an upper bound that is based on Mutual Information between the samples and its corresponding class [13]. In this case, the upper bound is inversely proportional to this statistic. We describe in section “**Mutual Information**” how we estimate this measure from the samples.

### Discriminant Analysis

Classic discriminant analysis techniques were often applied to linear feature extraction in order to find the projection matrix that preserves the class discriminability of data points. In this context, the class discriminability of the projected data is estimated from the data scatter in the projected space. We describe two of these measures, which are the ones we use in our work.

In Discriminant Analysis, two kind of statistics have been used for this purpose: *(i)* the within-class-scatter-matrix that shows the scatter of samples around the same class *S_W_*, and *(ii)* the between class scatter matrix *S_B_*.

The discriminability measure should be high when the between class scatter is high and the within class variation is low (samples from the same class are close among them and far from the other classes). Different analytic criteria have been proposed in the literature for this purpose, among we have chosen:
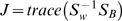
(1)


On the one hand, the first measure we consider is the discriminability criterion used in Fisher Linear Discriminant Analysis [8], that computes *S_B_* as
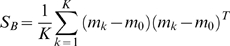
(2)where *m_K_* is the class-conditional sample mean and *m*_0_ is the unconditional (global) sample mean. Furthermore it estimates *S_W_* by
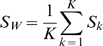
(3)where *S_k_* is the class-conditional covariance matrix for *C_k_* estimated from the data. We will denote this first measure by FLD.

On the other hand, Fukunaga and Mantock [10] proposed a non-parametric approach to compute the between class scatter matrix *S_B_*. In this case, the non-parametric between class scatter matrix is estimated as we describe following.

Let be *x* a data point in *X* with class label *C_j_*, and by *x^class^* class the subset of the *k* nearest neighbours of *x* among the data points in *X* with class labels different from *C_j_*. We calculate a local between-class matrix for *x* as:
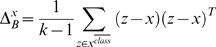
(4)


The estimate of the between-class scatter matrix *S_B_* is found as the average of the local matrices
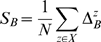
(5)


The resulting *S_B_* is used in the criterion [1], while *S_W_* remains as in the first case. We will denote this second discriminability measure by NDA.

### Mutual Information

The Mutual Information between two random variables *X* and *Y* is defined as:
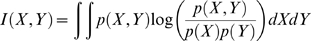
(6)where *p*(*X*) and *p*(*Y*) are their respective probability density functions. In this paper we compute mutual information between data points *X* and classes C. A large value of mutual information in this case means that we have much information about the class C given the observation *X*. On the other hand, if the mutual information is zero, then both variables are independent.

Notice that the computation of mutual information also necessitates the estimation of corresponding probability distributions. However, Torkkola [31] recently proposed a method which makes the computation of mutual information feasible by using a quadratic divergence measure that allows an efficient non-parametric implementation, without prior assumptions about class densities. In concrete, the Mutual Information from the data can be computed by
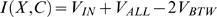
where
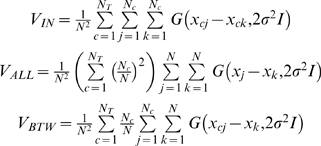
denoting a sample by one index, *x_i_*, if the class is irrelevant and by two indexes, *x_cj_*, when its class is relevant. The function *G* is a multi-dimensional Gaussian Kernel with covariance matrix Σ,
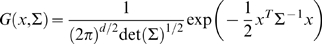
being *d* the corresponding dimensionality.

## References

[1] Atick J, Redlich A (1992). What does the retina know about natural scenes?. Neural Computation.

[2] Attneave F (1954). Some informational aspects of visual perception.. Psychological Review.

[3] Baddeley R, Abbott L, Booth M, Sengpiel F, Freeman T (1998). Responses of neurons in primary and inferior temporal visual cortices to natural scenes.. Proceedings of the Royal Society, London B.

[4] Barlow H, Rosenblith W (1961). Possible principles underlying the transformation of sensory messages.. Sensory Communication.

[5] Costen N, Parker D, Craw I (1994). Spatial content and spatial quantisation effects in face recognition.. Perception.

[6] Costen N, Parker D, Craw I (1996). Effects of high-pass and low-pass spatial filtering on face identification.. Perception and Psychophysics.

[7] Fiorentini A, Maffei L, Sandini G (1983). The role of high spatial frequencies in face perception.. Perception.

[8] Fisher R (1936). The use of multiple measurements in taxonomic problems.. Ann Eugenics.

[9] Fukunaga K (1990). Introduction to Statistical Pattern Recognition. 2nd ed.

[10] Fukunaga K, Mantock J (1983). Nonparametric discriminant analysis.. IEEE Transactions on Pattern Analysis and Machine Intelligence.

[11] Harris F (1978). On the use of windows for harmonic analysis with the discrete Fourier transform.. Proceedings of the IEEE.

[12] Hayes A, Morrone M, Burr D (1986). Recognition of positive and negative band-pass filtered images.. Perception.

[13] Hellman M, Raviv J (1970). Probability of error, equivocation and the Chernoff bound.. IEEE Transactions on Information Theory.

[14] Hosoya T, Baccus S, Meister M (2005). Dynamic predictive coding by the retina.. Nature.

[15] Howe C, Purves D (2002). Range image statistics can explain the anomalous perception of length.. Proceedings of the National Academy of Sciences USA.

[16] Keil M (2008). Does face image statistics predict a preferred spatial frequency for human face processing?. Proceedings of the Royal Society B.

[17] Laughlin S, de Ruyter van Steveninck R, Anderson J (1998). The metabolic cost of neural information.. Nature Neuroscience.

[18] Laughlin S, Sejnowski T (2003). Communication in Neural Networks.. Science.

[19] Lenny P (2003). The cost of cortical computation.. Current Biology.

[20] Levy W, Baxter R (1996). Energy-efficient neural codes.. Neural Computation.

[21] Linsker R (1988). Self-organization in a perceptual network.. IEEE Transactions on Computer.

[22] Long F, Yang Z, Purves D (2006). Spectral statistics in natural scenes predict hue, saturation, and brightness.. Proceedings of the National Academy of Sciences USA.

[23] Lotto R, Purves D (2000). An empirical explanation of color contrast.. Proceedings of the National Academy of Sciences USA.

[24] Lotto R, Williams S, Purves D (1999). Mach bands as empirically derived associations.. Proceedings of the National Academy of Sciences USA.

[25] Näsänen R (1999). Spatial frequency bandwidth used in the recognition of facial images.. Vision Research.

[26] Nundy S, Purves D (2002). A probabilistic explanation of brightness scaling.. Proceedings of the National Academy of Sciences USA.

[27] Ojanpää, Näsänen R (2003). Utilisation of spatial frequency information in face search.. Vision Research.

[28] Peli E, Lee E, Trempe C, Buzney S (1994). Image enhancement for the visually impaired: the effects of enhancement on face recognition.. Journal of the Optical Society of America A.

[29] Srinivasan M, Laughlin S, Dubs A (1982). Predictive coding: a fresh view of inhibition in the retina.. Proceedings of the Royal Society of London B.

[30] Tieger T, Ganz L (1979). Recognition of faces in the presence of two-dimensional sinusoidal masks.. Perception and Psychophysics.

[31] Torkkola K (2003). Feature extraction by non-parametric mutual information maximization.. J Mach Learn Res.

[32] Turk M, Pentland A (1991). Eigenfaces for Recognition,. Journal of Cognitive Neurosicence.

[33] Duda R, Hart PE, Stork DG (2000). Pattern Classification, 2nd ed.

[34] Hastie T, Tibshirani R, Friedman J (2001). The Elements of Statistical Learning.

[35] Fukunaga Keinosuke, Hung Sze-poR (1995). Bayes Error Estimation using Local Metrics. Electrical and Computer Engineering Technical Reports.. Purdue University School of Electrical and Computer Engineering.

[36] Xuan Guorong, Zhang Zhenping, Chai Peiqui, Shi YunQ, Fu Dongdong (2005). A Feature Selection Based on Minimum Upper Bound of Bayes Error.. IEEE 7th Workshop on Multimedia Signal Processing.

